# E3 ligase SMURF2 promotes adipogenesis and improves obesity complications by suppressing TGF-β signaling

**DOI:** 10.1016/j.jlr.2026.101061

**Published:** 2026-05-18

**Authors:** Xueqin Wu, Muchen Wu, Shiyu Du, Jiaxin Liu, Ruiping Wang, Yuhan Sun, Zhi Zheng, Junru Yang, Xiaowei Jia, Lulu Wang, Tao Lu, Chun Yang, Yan Gao, Dongliang Fang

**Affiliations:** 1 Department of Human Anatomy, School of Basic Medical Sciences, Capital Medical University, Beijing, China; 2 School of Basic Medical Sciences, Capital Medical University, Beijing, China; 3 General Surgery Department, Beijing Friendship Hospital, Capital medical University, Beijing, China; 4 Department of Gastroenterology, Beijing Ditan Hospital, Capital Medical University, Beijing, China; 5 Department of Experimental Center for Basic Medical Teaching, School of Basic Medical Sciences, Capital Medical University, Beijing, China; 6 Key Laboratory of Major Diseases in Children, Ministry of Education, Beijing, China

**Keywords:** Obesity, Metabolic Health, Adipogenesis, TGF-β/Smad Pathway, E3 Ligase SMURF2

## Abstract

Enhanced TGF-β/SMAD signaling causes adipose dysfunction by inhibiting adipogenesis and promoting adipocyte pathological hypertrophy in obesity development. Identifying novel regulators of the TGF-β pathway could significant important for modulating adipose metabolism. While ubiquitination of SMAD attenuates TGF-β signaling, it is still unknown whether this mechanism is involved in regulating adipose function. This study revealed that SMAD proteins undergo ubiquitination and degradation during the early stages of adipogenesis. E3 ligase SMURF2 was identified as the primary regulator in this biological process. *In vitro* studies demonstrated that SMURF2 deficiency significantly impaired adipogenic differentiation of preadipocytes, whereas SMURF2 overexpression markedly enhanced this process. In mouse studies, SMURF2 overexpression robustly promoted de novo adipogenesis, which in turn conferred resistance to high-fat diet-induced obesity and metabolic disorders. Furthermore, increased SMURF2 expression significantly improved and therapeutically ameliorated dysregulated glucose and lipid metabolism in obese mice. Interestingly, we designed a peptide to inhibit SMAD2 phosphorylation, thereby preventing its ubiquitination by SMURF2. Administration of this polypeptide conferred substantial metabolic benefits in obese mice. Our study uncovers a novel regulatory axis controlling adipose tissue expansion via the TGF-β/SMAD pathway. We anticipate that the findings from this project will provide new targets and insights for intervening in obesity and its metabolic complications.

Obesity has progressively become one of the most severe public health challenges. It is projected that over 4 billion people will be affected by obesity or overweight by 2035, accounting for 51% of the world's population (1). Obesity causes many chronic diseases in clinics and serves as a threat to human health (2). Obesity is primarily characterized by the excessive expansion of adipose tissue, driven by both hypertrophy of mature adipocytes and differentiation of preadipocytes into new adipocytes, which is termed adipogenesis (3, 4). During obesity progression, pathological hypertrophy of mature adipocytes leads to adipose tissue dysfunction, thereby inducing metabolic abnormalities such as insulin resistance and fatty liver disease. In contrast, adipogenesis is beneficial for maintaining adipose function by alleviating adipose inflammation, hypoxia, and fibrosis, which contributes to protecting against obesity complications (5, 6). The balance between adipocyte hypertrophy and adipogenesis during adipose expansion is crucial for systemic metabolic health. Identifying targets in this balance holds significant value for theory and application in combating obesity.

The transduction of the TGF-β signaling pathway is initiated by ligand binding to type II and type I receptors, forming a complex that induces phosphorylation of the type I receptor by the type II receptor. The activated type I receptor then recruits and phosphorylates downstream SMAD2/3, which subsequently form a complex with SMAD4 and translocate to the nucleus to function as transcription factors. It is well established that TGF-β/SMAD signaling activity is significantly enhanced during obesity. This pathway contributes to adipose tissue dysfunction by suppressing adipogenesis and promoting pathological hypertrophy of adipocytes. Studies have shown that TGF-β potently inhibits adipogenesis, and overexpression of TGF-β leads to lipodystrophic diabetes and fatty liver in mice (7, 8). Mechanistically, TGF-β inhibits the formation of the PPARγ-C/EBPα complex via SMAD3 to limit adipogenesis (9). Furthermore, enhanced TGF-β signaling during obesity promotes adipocyte hypertrophy, exacerbating adipose inflammation and fibrosis, which subsequently leads to systemic metabolic dysregulation. Thus, the TGF-β pathway plays a critical role in regulating adipose remodeling and metabolic adaptation during obesity. Exploring potential targets in the TGF-β pathway might hold significant therapeutic value.

The activity of the TGF-β signaling pathway is finely regulated at the levels of SMAD transcription, translation, and post-translational modifications (10, 11). SMURF (SMAD ubiquitination regulatory factor) and WW-HECT family are classical E3 ubiquitin ligases of SMAD ubiquitination. Additionally, novel regulatory mechanisms involving SMAD ubiquitination and deubiquitination continue to be discovered. For instance, NEDD4L alleviates fibrosis by promoting ubiquitin-mediated degradation of phosphorylated SMAD3 in pulmonary fibrosis (12). In hepatocellular carcinoma, PJA1 induces SMAD3 ubiquitination and inhibits TGF-β signaling to promote tumor progression (13). Activated SMAD2/3 could be deubiquitinated by OTUB1, leading to increased protein stability, which plays an important role in modulating TGF-β signaling (14). In melanoma studies, it is shown that acetylated SMAD3 is more stable to promote tumor growth (15). These studies indicate that ubiquitination of SMAD proteins is closely associated with the transduction of TGF-β signaling and serves as a critical target for controlling this pathway. However, the regulation of SMAD ubiquitination in the context of adipose metabolism remains unclear.

Ubiquitination is characterized by specificity and reversibility, offering potential for clinical drug development. In recent years, research on ubiquitination in metabolic diseases has attracted increasing attention of researchers (16, 17, 18). E3 ligase APPBP2 inhibits beige fat formation by regulating PRDM16 protein stability, which is the core regulator of beige adipogenesis, thereby promoting obesity and diabetes (19). Additionally, PPARδ is a key molecule promoting mitochondrial oxidative phosphorylation and energy expenditure. The deubiquitinase OTUD3 stabilizes PPARδ and enhances its function, exerting protective effects against obesity and diabetes (20). Therefore, this study aims to further investigate novel ubiquitination-related regulatory mechanisms closely associated with systemic metabolism, providing potential intervention targets for clinical application.

This study demonstrates that E3 ligase SMURF2 promotes adipogenesis by regulating SMAD2/3 ubiquitination in a series of both in vitro and in vivo experiments, which is beneficial for mitigating metabolic complications induced by obesity. This mechanism represents a key aspect of TGF-β-mediated regulation of adipose physiological function. Inhibition of SMAD phosphorylation with small molecule peptides to prevent ubiquitination by SMURF2 confers significant metabolic benefits in obese mice. This study reveals a bypass regulatory mechanism of the TGF-β/SMAD pathway in adipose expansion, offering a novel method for modulating adipose homeostasis. We anticipate that this study will provide novel insights for the intervention of obesity and metabolic disorders.

## Materials and Methods

### Patient studies

Clinical tissue samples were collected from Friendship Hospital of Capital Medical University, involving 76 patients with HNSCC admitted between September 2016 and September 2018. The tissues were rinsed with PBS and promptly stored at −80 °C. Written informed consent was obtained from all patients prior to participation. The study was approved by the Institutional Review Board of Jeonbuk National University Hospital (2024-P2-097-02) and performed in accordance with the ethical standards of the Declaration of Helsinki.

### Mouse models

All male C57BL/6J mice, aged 6–8 weeks, used in this study were purchased from Beijing Vital River Laboratory Animal Technology Co., Ltd. The mice were housed in a facility with controlled temperature (22 ± 2°C) and humidity (40%–60%), maintained on a regular 12:12 light/dark cycle, and provided with rodent chow and free access to water. All animal experiments occurred in the animal facility of Capital Medical University and received approval from the Animal Use and Care Committee. For high-fat diet (HFD) feeding experiments, the 6–8-week-old male mice were fed HFD (60% kcal, Research Diets, D12492) for a duration of 12–20 weeks, as indicated in figure legends. All animal experiments were approved by the Medical Ethics Committee of Capital Medical University.

### Administration of adeno-associated viral (AAV) vectors

AAV9-SMURF2 was employed to overexpress SMURF2 in white adipose tissue (WAT), while AAV9-GFP served as the control (WeiZhen Biosciences, Shanghai, China). Subcutaneous WAT (sWAT) pads on both sides of the mice received the AAV injections. The mice were anesthetized with ketamine (100 mg/kg) and xylazine (10 mg/kg). For AAV delivery in sWAT, longitudinal incisions were made in the skin around the inguinal regions, followed by the exposure of the fat pad using tweezers. AAV was injected into each fat pad at multiple sites (8–10 sites per fat pad). The total injection volume was 50 μl, with a total viral titer of 1 × 10^11^ viral genomes (vg) for each pad. For AAV delivery in epididymal WAT (eWAT), a laparotomy was performed to expose the eWAT, and the AAV was injected as previously described. The abdomen was then rinsed with sterile saline solution and closed with a two-layer suture.

### Peptide treatment

In the high-fat diet (HFD)-induced obese mouse model, 6- to 8-week-old male mice received HFD (60 kcal%, Research Diets, D12492) for 12 weeks. Subsequently, the mice were divided into two groups and intraperitoneally injected with either normal saline (vehicle) or P1 peptide (GRKKRRQRRRPQ-GG-RRLAAXSMDTGL, GRKKRRQRRRPQ is the cell penetrating peptide (CPP) part, RRLAAXSMDTGL is the key region for targeted inhibition of SMAD2 phosphorylation, GG is two glycine (Gly) as flexible linkers to avoid the spatial conformation of transmembrane peptides affecting the binding of downstream inhibitory sequences to kinases) from Syneptide Co., Ltd. (Nanjing, China) at a dosage of 8 mg/kg body weight three times per week for 6 weeks. Glucose tolerance and insulin tolerance tests were conducted in the fifth and sixth week following treatment, respectively.

### Cell culture and adipogenic differentiation

3T3-L1 cells were cultured in high-glucose DMEM supplemented with 10% FBS and 1% penicillin/streptomycin at 37°C in an atmosphere of 5% CO_2_ and 95% air. The day on which the cells reached confluence was designated as day −2. Two days after confluence, referred to as day 0, the cells underwent differentiation induction using DMEM that contained 10% FBS, 1% penicillin/streptomycin, 5 μg/ml insulin, 0.5 mmol/L 3-isobutyl-1-methylxanthine, and 1 mmol/L dexamethasone for 2 days (day 2). Following this induction, the cells were maintained in culture medium supplemented with 5 μg/ml insulin for an additional 2 days. After the induction of adipogenic differentiation, the cells were sustained in DMEM containing 10% FBS and 1% penicillin/streptomycin for 4 days, with medium changes occurring every 2 days. Additionally, the cells were pretreated with MG132 at a concentration of 20 μM for 2 h.

### Primary SVF isolation

For stromal vascular fraction (SVF) isolation, epididymal white adipose tissue (ewat) from 8-week-old mice was digested with collagenase I (Worthington, 41J21480) in 1× PBS at 37 °C for 60 min. The resulting cell suspension was centrifuged, resuspended, and sequentially filtered through 80-mesh and 40-μm cell strainers. Cells were then centrifuged and plated in 6-well plates. Cells were cultured in DMEM/F12 supplemented with 10% FBS and 1% penicillin/streptomycin at 37 °C in a humidified atmosphere containing 5% CO_2_.

### Plasmid construction and cell transfection

For stable RNA interference, lentiviruses were obtained from Beijing Likely Biotechnology Co., Ltd (Beijing, China). Plasmids encoding SMURF2 were constructed by Beijing Likely Biotechnology Co., Ltd. The plasmid encoding HA-UB was kindly provided by Dr Ping Xie at Capital Medical University. siRNA targeting mouse NEDD4L and negative control siRNA (siNC) were purchased from Beijing Likely Biotechnology Co., Ltd. For cell transfection, the transient expression of genes was achieved using Lipofectamine with Plus reagents, following the manufacturer's instructions. Transient siRNA transfection into SVF was conducted in vitro using Lip2000, in accordance with the manufacturer's guidelines.

### Real-time RT-PCR

Total RNA was extracted from tissues or cells using TRIzol reagent (Takara Bio) following the manufacturer's instructions and transformed into cDNA utilizing a cDNA synthesis kit (Vazyme). The RNA concentration ranged from 1500 to 3000 ng/μl as assessed by NanoDrop2000. Real-time PCR analysis was conducted with SYBR Green Master Mix (Vazyme) in a CFX96 Real-time System, C1000 Thermal Cycler (Bio Rad). The primer sets we used were as Supplementary Material Table S1.

### Western Blot and immunoprecipitation

Protein samples were isolated from tissue or cell samples using RIPA lysis buffer (65 mM Tris-HCl, pH 7.5, 150 mM NaCl, 1 mM EDTA, 1% NP-40, 0.5% sodium deoxycholate, 0.1% SDS, protease inhibitor cocktail tablets (P1265-2, Applygen, Beijing, China), and phosphatase inhibitor tablets (P1260-5, Applygen). Protein concentrations were measured with the BCA Protein Assay Kit (23225, Thermo Fisher Scientific). Subsequently, proteins were separated on 10% SDS-PAGE gels and transferred onto PVDF membranes (IPVH00010, Millipore). Following blocking in 5% skim milk, the membranes were incubated overnight at 4°C with primary antibodies and then for 1 h at room temperature with the corresponding secondary antibodies. The primary antibodies we used were as Supplementary Material Table S2. After three washes in TBST, the immune complexes were detected using ECL detection reagents (SQ201, Epizyme). Protein expression levels were quantified with Image Lab software and normalized to the levels of HSP90, which served as a loading control. Immunoprecipitation was performed using the Pierce™ IP kit (26146, Thermo) according to the manufacturer's guidelines. The antibodies used in the study are as follows.

**Table:** 

Smurf2 (D8B8)	12024	CST
Smad2 Rabbit mAb	19114	ABclonal
Phospho-Smad2-T220 Rabbit pAb	AP0909	ABclonal
Phospho-Smad3-T179 Rabbit pAb	AP0554	ABclonal
Smad3 Rabbit pAb	(A16913)	ABclonal
SMAD4 (D3R4N)	46535	CST
PPARγ(81B8)	71433	CST
C/EBPα(PT0665R)	YM8474	Immunoway
C/EBPβ	YT0553	Immunoway
TGFβ R1	sc-101574	SANTA
TGFβ R11	sc-17792	SANTA
HSP90	13171-1-AP	Proteintech
GAPDH	5174	CST
Rabbit Anti-Mouse IgG	ab6728	abcam
Goat Anti-Rabbit IgG	ab6721	abcam

### Ubiquitination and cycloheximide chase assay

3T3-L1 cells were co-transfected with HA-ubiquitin. Prior to harvest, cells were treated with 200 pM TGF-β1 for 1 h and 20 μM MG132 for 4 h. Cells were lysed in IP lysis buffer, and immunoprecipitation was carried out using Protein G magnetic beads (HY-K0204, MedChemExpress) following the manufacturer’s instructions. Immunoprecipitates were boiled in SDS loading buffer and analyzed by Western blotting with an anti-HA antibody. For protein stability assays, cells were treated with 100 μg/ml CHX for the indicated time points.

### Histology and oil red O staining

Upon the sacrifice of animals, the WAT and liver tissues were dissected and immediately fixed in 4% paraformaldehyde. The tissues underwent routine processing for paraffin embedding, and 5 μm sections were cut and mounted on glass slides. For hematoxylin-eosin staining and immunohistochemistry (IHC), the liver, subcutaneous white adipose tissue (sWAT), and epididymal white adipose tissue (eWAT) were fixed overnight in 4% formalin, embedded in paraffin, and sectioned into 5 μm thick slices. Immunohistochemistry was performed on paraffin-embedded WAT and liver sections. Antigen retrieval occurred in citrate buffer using a pressure cooker. IHC analysis utilized an IHC detection kit (PV-9001, ZSGB-Bio) according to the manufacturer's guidelines. The sections were visualized with DAB (ZLI-9018; ZSGB-Bio, China) and counterstained with hematoxylin. Differentiated 3T3-L1 cells were fixed with 4% formaldehyde in PBS for 30 min and subsequently washed three times in PBS for 10 min each. For Oil-Red-O staining, the cells were stained with 0.3% Oil Red O for 30 min in the dark at room temperature, followed by three washes in PBS for 10 min. Oil-Red-O staining for lipid accumulation was directly imaged using an inverted microscope, or lipid content was quantified after Oil-Red-O extraction with isopropanol by measuring light absorbance at 490 nm.

### Glucose and insulin tolerance test

Glucose tolerance was assessed in 16-h fasted mice by intraperitoneally administering glucose (1.5 g/kg of body weight). Blood glucose levels were monitored from the tail before and 15, 30, 60, 90, and 120 min post glucose and insulin injections. Insulin sensitivity was evaluated in 6-h fasted mice by intraperitoneally administering insulin (0.75U/kg of body weight). Blood glucose levels were measured from the tail before and 15, 30, and 60 min after glucose and insulin injections. Blood glucose levels were determined using a Sinocare glucometer on tail-vein blood samples at specified time points post administration.

### Biochemical analysis

Serum levels of TG (A110-1), TC (A111-1), FFA (A042-2-1), ALT (C009-3-1), and AST (C010-3-1), as well as hepatic TG levels, were measured following the instructions provided by biochemical assay kits from Nanjing Jiancheng Bioengineering Institute.

### Statistical analysis

All data were analyzed using different statistical methods as shown in figure legends. The *P* values were also indicated in figures and captions. Each experiment was repeated independently with similar results at least three times except for particular instructions.

## Results

### Identification of SMAD2/3 complex ubiquitination regulated by E3 ligase SMURF2 in adipogenesis

**Fig. 1:**
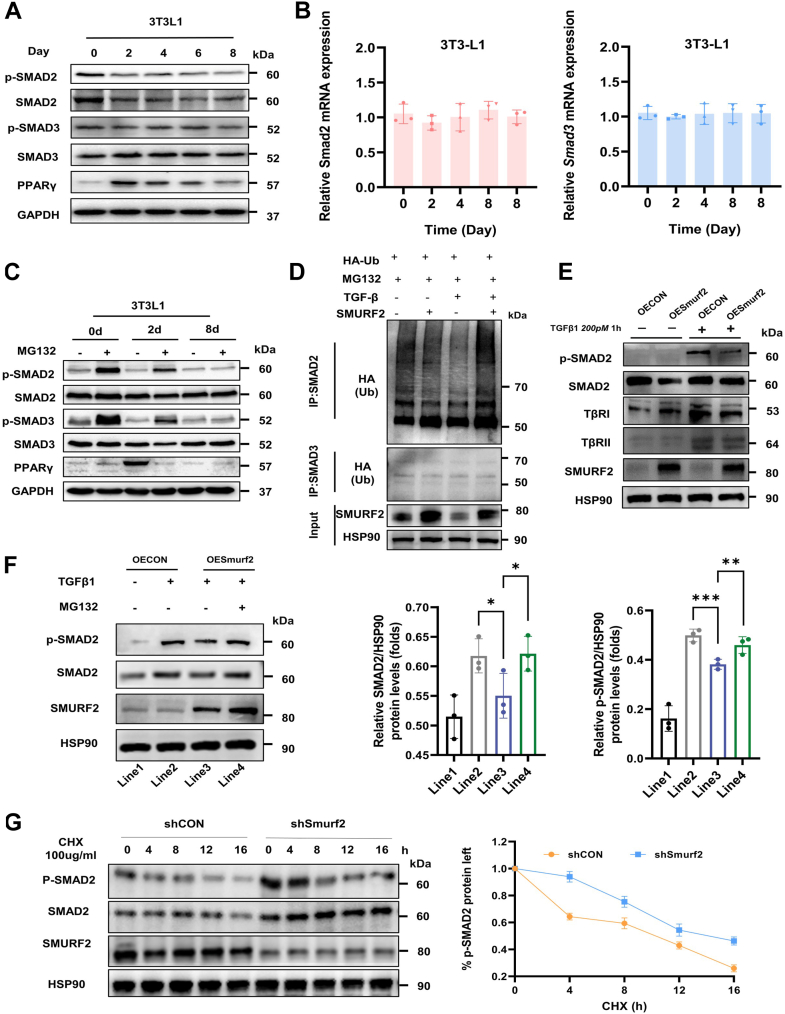
E3 Ligase SMURF2 Mediates SMAD2/3 Ubiquitination and Degradation During Early Adipogenesis. A-B: SMAD2/3protein (A) and mRNA (B) levels during adipogenic differentiation of 3T3-L1 cells at days 0, 2, 4, 6 and 8, analyzed by Western blot and qRT-PCR. C: Western blot analysis of SMAD2/3 protein levels during adipogenic differentiation of 3T3-L1 cells at day 0, 2, and 8, cells were treated with 20 μM MG132 for 4h before harvest. D: 3T3-L1 cells were co-transfected with HA-ubiquitin and either control vector or SMURF2. Cells were treated were incubated without or with 200 pM TGFβ1 for 1 h and 20 μM MG132 treatment for 4 h. Cell lysates were separately immunoprecipitated with anti-SMAD2 or anti-SMAD3 antibodies, and ubiquitinated SMAD2/SMAD3 was detected by immunoblotting with anti-HA. Input lysates were immunoblotted with anti-SMURF2 and anti-HSP90. E: Protein levels of p-SMAD2/SMAD2, TβRI, and TβRII in 3T3-L1 cells with SMURF2 overexpression, cells were treated with vehicle or 200 pM TGFβ1 for 1 h before harvest. F: Western blot and protein fold change of p-SMAD2 and SMAD2 in 3T3-L1 cells with CON or SMURF2 overexpression, cells were incubated with or without 200 pM TGFβ1 for 1 h or 20 μM MG132 treatment for 4 h. G: p-SMAD2 and SMAD2 protein levels in 3T3-L1 cells with SMURF2 knockdown, followed by treatment with the 100 μg/ml CHX. For statistical analysis, data are presented as mean ± SEM. One-way ANOVA tests were performed in B, F. Two-way ANOVAs test was performed in G. ∗*P* < 0.05, ∗∗*P* < 0.01, ∗∗∗*P* < 0.001, n = 3 biological replicates.

To explore the role of SMAD ubiquitination in adipogenesis by regulating TGF-β signaling. We performed adipogenic differentiation of 3T3-L1 preadipocytes, it was shown that both p-SMAD2 and total SMAD2 were significantly reduced at 2 days of differentiation. p-SMAD3 was slightly downregulated, and SMAD3 showed no significant change (Fig. 1A). Both *Smad2* and *Smad3* mRNA levels remained unaltered during adipogenic differentiation (Fig. 1B). Given that ubiquitination is a key post-translational modification for protein degradation, we investigated whether SMAD reduction is associated with proteasome-dependent degradation using the proteasome inhibitor MG132. Strikingly, MG132 treatment remarkably preserved the p-SMAD2/3 protein levels in 3T3L1 cells at 0 days and 2 days for differentiation but not at 4 days, 6 days, and 8 days (Fig. 1C and Supplemental Fig. S1A). This suggests that SMAD protein abundance dropped in the early period of adipogenic differentiation is closely related to the ubiquitin-proteasome system. While NEDD4L has been reported to regulate the ubiquitination of p-SMAD2/3 in other cell types (21, 22, 23), our data showed that knockdown of NEDD4L in 3T3-L1 cells does not alter the protein levels of p-SMAD2/3 (Supplemental Fig. S1B), nor affect the expression of adipogenic genes (Supplemental Fig. S1C). These findings indicate that NEDD4L is dispensable for SMAD2/3 ubiquitination in adipocytes. As SMURF2 is another classical E3 ligase for SMAD (24, 25), we tried to explore its role in this adipogenic process. Ubiquitination assay showed that SMURF2 mainly targets TGF-β-induced poly-ubiquitination of p-SMAD2 but not p-SMAD3/SMAD3 (Fig. 1D). Further, we found that SMURF2 overexpression in 3T3-L1 cells obviously decreased the TGF-β-induced p-SMAD2 and slight decrease in total SMAD2 level, while TβRII and TβRI levels remained unchanged (Fig. 1E). Conversely, SMURF2 knockdown significantly increased the protein level of p-SMAD2 and slightly decreased the SMAD2 level (Supplemental Fig. S1D). Meanwhile, SMURF2 knockdown didn’t affect TβRII and TβRI protein levels. The reduction in p-SMAD2/SMAD2 protein levels caused by SMURF2 overexpression was rescued by MG132 treatment (Fig. 1F). Cycloheximide (CHX) chase experiment showed that SMURF2 deficiency significantly prolonged the half-life of p-SMAD2 (Fig. 1G). Collectively, these results identify that the E3 ligase SMURF2-mediated SMAD ubiquitination plays a role in the biological process of adipogenesis.

### SMURF2 promotes adipogenic differentiation in vitro

**Fig. 2:**
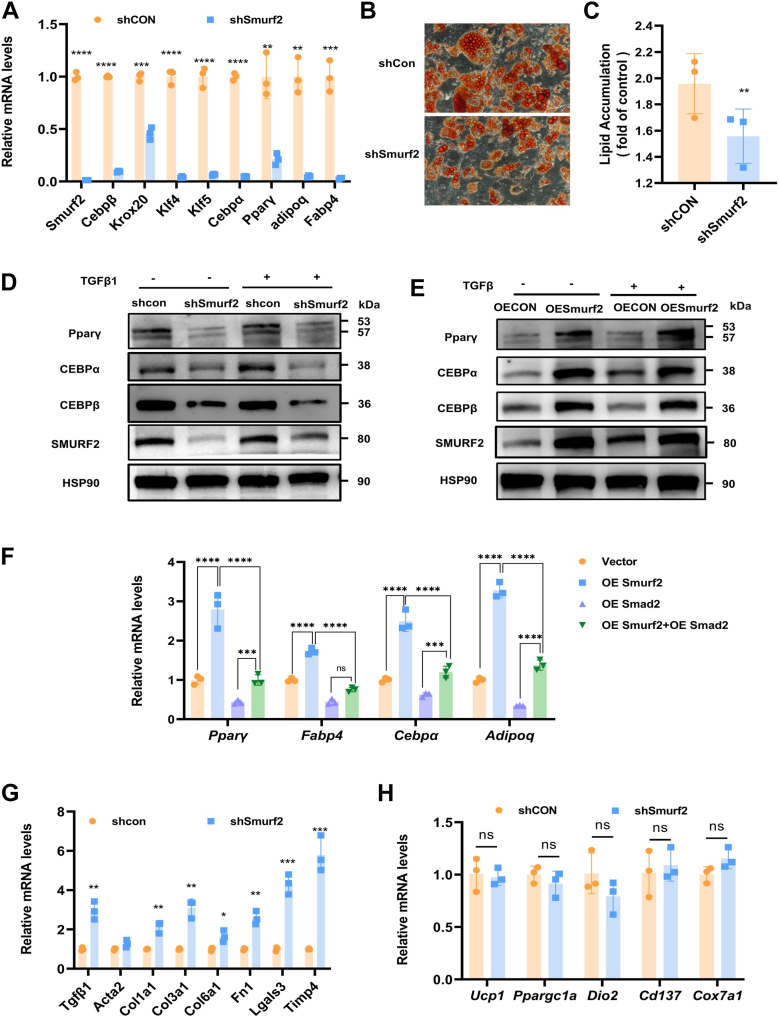
SMURF2 Promotes Adipogenic Differentiation of Preadipocytes *in Vitr*o. A: mRNA levels of adipogenic genes in 3T3-L1 cells with *Smurf2* knockdown on day 6 of adipocyte differentiation. B: Oil Red O staining for assessment of lipid accumulation in 3T3-L1 cells with *Smurf2* knockdown on day 8 of adipocyte differentiation (×20). C: Quantification of oil red O staining. D-E: Protein levels of adipogenic regulators in 3T3-L1 cells with *Smurf2* knockdown (D) or overexpression (E) on day 6 of white adipocyte differentiation, with or without TGF-β treatment, analyzed by Western blot. F: mRNA levels of adipogenic genes in 3T3-L1 cells on day 6 of adipocyte differentiation after *Smurf2* overexpression and/or *Smad2* overexpression. G: mRNA levels of fibrosis genes of 3T3-L1 cells after *Smurf2* knockdown with TGFβ1 treatment (200 pM, 1 h), analyzed by qRT-PCR. H: mRNA levels of thermogenic genes of 3T3-L1 cells after *Smurf2* knockdown. For statistical analysis, data are presented as mean ± SEM. Unpaired t-tests were performed in A, B, G, H. One-way ANOVA test was performed in F. ∗*P* < 0.05, ∗∗*P* < 0.01, ∗∗∗*P* < 0.001, ∗∗∗∗*P* < 0.0001. n = 3.

Next, we studied the function of SMURF2 in regulating adipogenic differentiation. We found that SMURF2 expression is upregulated at 2days differentiation of 3T3-L1 cells (Supplemental Fig. S2A). It was shown that SMURF2 is enriched in adipocyte progenitors but not the mature cells (Supplemental Fig. S2B). During adipogenic differentiation, SMURF2 knockdown significantly inhibited adipogenic genes expression (Fig. 2A). Conversely, SMURF2 overexpression upregulated adipogenic genes level (Supplemental Fig. S2C). Decreased lipid accumulation was observed in differentiated adipocytes with SMURF2 knockdown by Oil Red O staining (Fig. 2B, C). SMURF2 deletion also inhibited the protein levels of PPARγ, C/EBPα and C/EBPβ in adipocytes with or without TGFβ treatment (Fig. 2D). Conversely, SMURF2 overexpression increased the PPARγ,C/EBPα and C/EBPβ protein levels (Fig. 2E). A rescue study by overexpressing *Smad2* in *Smurf2*-overexpressing 3T3L1 cells showed that the upregulation of adipogenic genes by *Smurf2* overexpression was significantly inhibited with *Smad2* overexpression (Fig. 2F). Besides, SMURF2 knockdown increases the expression of fibrotic genes including *Tgfβ1, Col1a1, Col3a1, Fn1,* and *Timp4* (Fig. 2G), whereas SMURF2 overexpression suppresses these fibrotic genes expression (Supplemental Fig. S2D). We also studied the regulation of beige adipogenic differentiation by SMURF2 knockdown and overexpression. It was shown that thermogenic genes expression was not altered by SMURF2 knockdown or overexpression (Fig. 2H, Supplemental Fig. S2E). Together, these data show that SMURF2 plays an important role in driving adipogenic process *in vitro*.

### SMURF2 overexpression promotes adipogenesis in mice

**Fig. 3:**
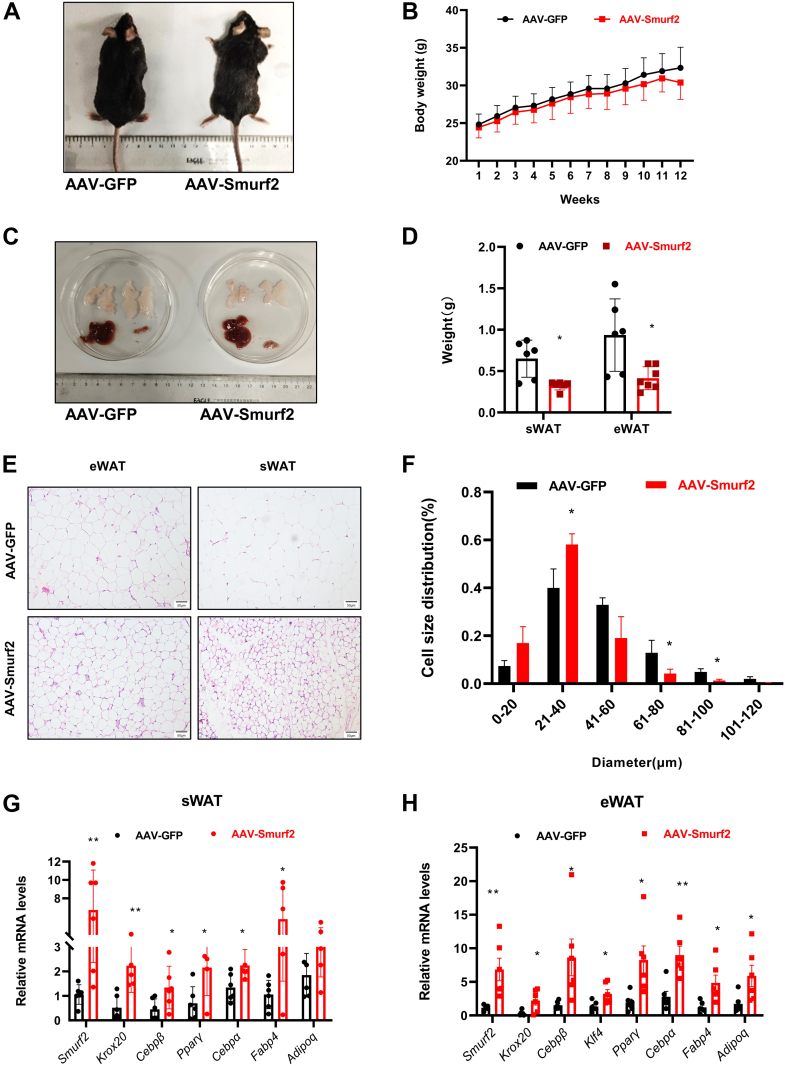
SMURF2 Overexpression Enhances Adipogenesis in Mice. A: Representative photographs of AAV-GFP and AAV-Smurf2 mice fed with NCD for 12 weeks. n = 8. B: Body weight of AAV-GFP and AAV-Smurf2 mice. n = 8. C, Representative photographs of subcutaneous adipose tissue (upper left), liver (lower left), epididymal adipose tissue (upper right) and brown adipose tissue (lower right) from AAV-GFP and AAV-Smurf2 mice. n = 8. D: Fat tissue weight of AAV-GFP and AAV-Smurf2 mice fed with NCD for 12 weeks. n = 6. E: HE staining of sWAT, eWAT in AAV-GFP and AAV-Smurf2 mice, scale bar, 50 μm. F: Statistical analysis of adipocytes’ number and size in eWAT. G–H: mRNA levels of adipogenic genes in sWAT (G) and eWAT (H) of AAV-GFP and AAV-Smurf2 mice. n = 6. For statistical analysis, data are presented as mean ± SEM. Two-way ANOVAs was performed in B. Unpaired t-tests were performed in D, F, G, H. ∗*P* < 0.05, ∗∗*P* < 0.01.

We next studied the role of SMURF2 in mouse adipogenesis based on our in vitro findings. An adeno-associated virus (AAV) vector was constructed and injected in local adipose to overexpress SMURF2 in mice. Validation of SMURF2 overexpression in WAT but not in liver and skeletal muscle was confirmed (Fig. 3G, H, Supplemental Fig. S3A). With a normal chow diet for 12 weeks, a slight lower body weight was recorded in SMURF2 overexpression group (Fig. 3A, B). SMURF2 overexpression caused a decrease in fat mass of subcutaneous white adipose tissue (sWAT) and epididymal white adipose tissue (eWAT), (Fig. 3C, D). Histological analysis showed smaller adipocytes in both eWAT and sWAT of SMURF2 overexpression mice (Fig. 3E, F). These phenotypic traits related to body weight and fat physiology appear to be closely attributed to the role of TGF-β in inhibiting adipogenesis and promoting hypertrophy. mRNA levels of adipogenic genes were elevated in both sWAT and eWAT of SMURF2 overexpressing mice (Fig. 3G, H). Serum levels of FFA and T-CHO were reduced in SMURF2-overexpressing mice, while TG levels remained unchanged (Supplemental Fig. S3, B–D). Thermogenic gene expression in iWAT was comparable between the SMURF2 overexpression and control group (Supplemental Fig. S3E). Overall, these results indicate that SMURF2 overexpression drives adipogenesis in vivo, and we propose a role of SMURF2 in obesity development while TGF-β activity is enhanced.

### SMURF2 level is inversely correlated with obesity and its overexpression improves obesity-induced metabolic dysfunctions

**Fig. 4:**
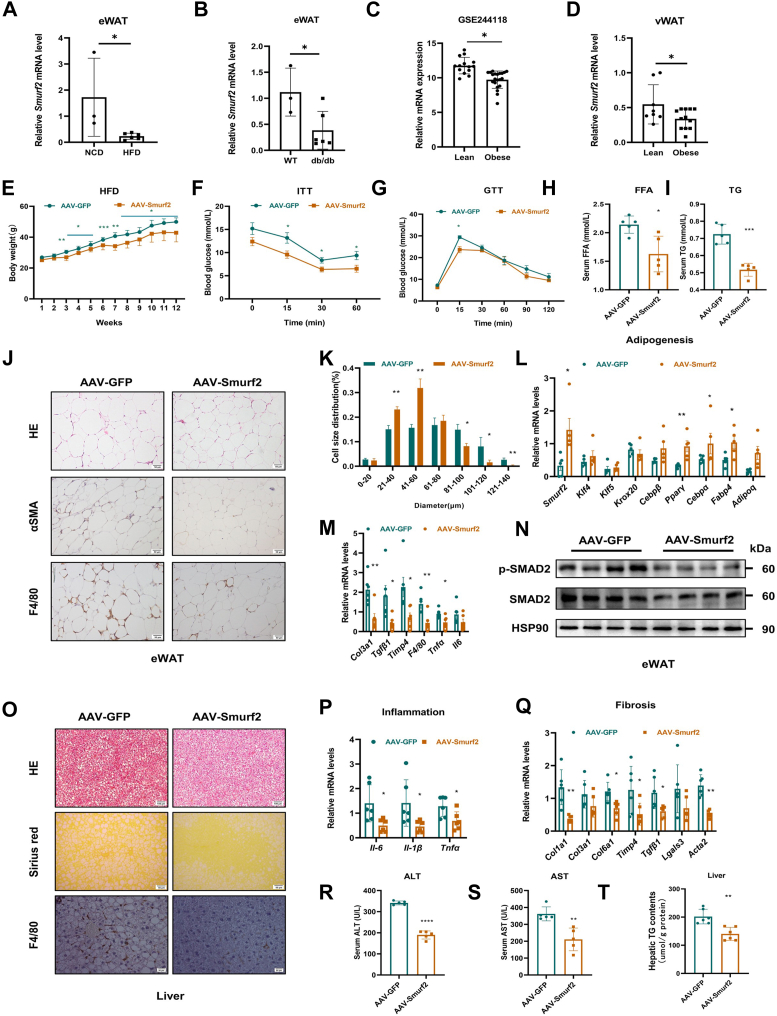
A, B: qRT-PCR analysis of *Smurf2* mRNA levels in epididymal WAT (eWAT) of mice fed a high-fat diet (HFD) vs. chow diet (A), and in db/db vs. WT mice (B). C, D, Analysis of *SMURF2* mRNA expression in human subcutaneous WAT (sWAT) from public datasets (GSE244118) (C) and in an independent cohort of vWAT samples from lean and obese individuals (D) E–T: Male C57BL/6J mice were injected with AAV-GFP or AAV-Smurf2 into sWAT and eWAT and then fed an HFD for 12 weeks. E: Body weight curve of AAV-GFP or AAV-Smurf2 mice during 12 weeks HFD feeding. n = 8. F, G: GTT (F) and ITT (G) after 12 weeks HFD feeding. n = 8. H, I: Serum levels of FFA (H) and TG (I) of the mice. n = 6. J: Representative H&E, αSMA, and F4/80 staining of eWAT sections of mice. Scale bar, 50 μm. K: Statistical analysis of adipocyte size in (J). L, M: qRT-PCR analysis of adipogenic genes (L) and profibrotic/proinflammatory genes (M) expression in eWAT. n = 6. N: Western blot analysis of protein levels of p-SMAD2 and SMAD2 in eWAT, n = 4. O: Representative H&E (scale bar, 100 μm), Sirius red (scale bar, 50 μm), and F4/80 (scale bar, 20 μm) staining of liver sections of mice. P, Q: qRT-PCR analysis of hepatic inflammatory (P) and fibrotic (Q) genes expression. n = 6. R, S: Serum levels of ALT (R) and AST (S) of mice. n = 6. T: Hepatic TG levels in mice. n = 6. For statistical analysis, data are presented as mean ± SEM. Two-way ANOVAs was performed in (E–G). Unpaired t-tests were performed in (A–D, H, I, K, L, P–T). ∗*P* < 0.05, ∗∗*P* < 0.01, ∗∗∗*P* < 0.001, ∗∗∗∗*P* < 0.0001. AAV, adeno-associated virus; ALT, alanine aminotransferase; AST, aspartate aminotransferase; FFA, free fatty acid; GTT, glucose tolerance test; ITT, insulin tolerance test; SEM, standard error of the mean; TG, triglyceride; vWAT, visceral WAT; WT, wild-type.

Based on our findings that SMURF2 drives adipose metabolic remodeling (Fig. 3), we then focused on its role in obesity development. In eWAT of high-fat diet (HFD)-induced obese mice, *Smurf2* mRNA level was significantly lower in eWAT and sWAT compared to chow diet fed controls (Fig. 4A and Supplemental Fig. S4A). Similarly, its expression is also reduced in eWAT and sWAT of db/db mice (Fig. 4B and Supplemental Fig. S4B). In human datasets (GSE152991 and GSE244118), it is revealed that *SMURF2* level is significantly lower in sWAT of individuals with obesity compared to lean controls (Fig. 4C and Supplemental Fig. S4C). Consistently, *SMURF2* mRNA levels were markedly reduced in both visceral white adipose tissue (vWAT) and sWAT from obese individuals relative to lean subjects (Fig. 4D and Supplemental Fig. S4D).

To identify the role of SMURF2 in obesity development and related metabolic disease. We constructed the SMURF2 overexpressing mice model with AAV administration as mentioned above. It was shown that AAV-Smurf2 mice gained less body weight than controls with HFD feeding (Fig. 4E and Supplemental Fig. S4E). Reduced fat mass and liver weight were detected in SMURF2 overexpressing mice (Supplemental Fig. S4F, G). Moreover, the HFD-fed AAV-Smurf2 mice showed improved glucose and insulin tolerance compared with the control (Fig. 4F, G). Serum levels of free fatty acids (FFA), triglycerides (TG), and total cholesterol (T-CHO) were also lower in SMURF2 overexpressing mice (Fig. 4H, I and Supplemental Fig. S4H). Histological analysis showed a higher ratio of smaller adipocytes in eWAT and sWAT from HFD-fed AAV-Smurf2 mice compared to controls (Fig. 4J, K and Supplemental Fig. S4I). This was accompanied by reduced F4/80^+^ macrophage infiltration and diminished αSMA^+^ fibrotic visions (Fig. 4J and Supplemental Fig. S4I). Adipogenic genes were upregulated (Fig. 4L and Supplemental Fig. S4J), whereas fibrotic genes and inflammatory genes were downregulated in both sWAT and eWAT of SMURF2 overexpression mice (Fig. 4M and Supplemental Fig. S4K, L). Meanwhile, SMURF2 overexpression didn’t affect the expression of thermogenic genes in sWAT of mice (Supplemental Fig. S4M). SMURF2 overexpression significantly reduced the level of p-SMAD2 in eWAT of mice (Fig. 4N), confirming the inhibition of TGF-β signaling.

We also studied the liver physiology in SMURF2 overexpressing obese mice. Histological analysis showed reduced hepatic lipid droplet accumulation, decreased F4/80 and Sirius Red content in AAV-Smurf2 mice compared to control (Fig. 4O). Inflammatory genes and fibrotic genes were also downregulated in the liver (Fig. 4P, Q). Serum levels of ALT and AST were significantly lower in AAV-Smurf2 mice (Fig. 4R, S). TG content in the liver was lower in SMURF2 overexpressing mice (Fig. 4T). Together, these findings show that adipose SMURF2 overexpression prevents diet-induced metabolic dysfunction and hepatic injury through enhanced adipogenesis.

### SMURF2 overexpression in obese mice alleviates metabolic dysfunctions

**Fig. 5:**
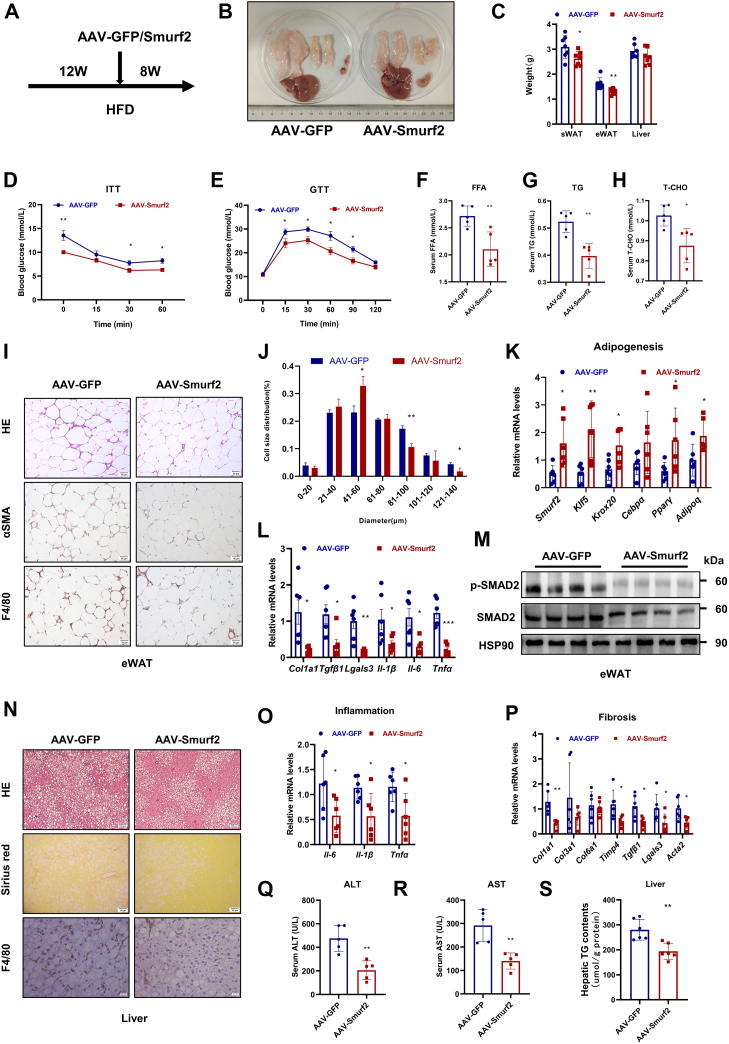
SMURF2 Overexpression Improves Metabolic Health of Obese Mice. A: Schematic diagram of animal model, mice were injected with AAV-GFP or AAV-Smurf2 into sWAT and eWAT after 12 weeks HFD feeding and then for an additional 8 weeks HFD. B: Representative photographs of subcutaneous adipose tissue (upper left), liver (lower left), epididymal adipose tissue (upper right) and brown adipose tissue (lower right) from AAV-GFP and AAV-Smurf2 mice. n = 8. C: Tissue weight of AAV-GFP and AAV-Smurf2 mice. n = 6. D–E: GTT (D) and ITT (E), n = 6. F–H: Serum levels of FFA (F), TG (G), and T-CHO (H) of the mice, n = 6. I, Representative H&E, F4/80, and αSMA staining of eWAT sections of the mice, Scale bar, 50 μm. J: Statistical analysis of adipocyte size in (I). K–L: qRT-PCR analysis of adipogenic genes (K) and pro-fibrotic/pro-inflammatory genes expression (L) in eWAT of the mice, n = 6. M: Western blot analysis of protein levels of p-SMAD2 and SMAD2 in eWAT of the mice, n = 4. N, Representative H&E (scale bar, 100 μm), Sirius red (scale bar, 50 μm), and F4/80 (scale bar, 20 μm) staining of liver sections of mice. O-P: qRT-PCR analysis of hepatic inflammatory (O) and fibrotic (P) genes expression. n = 6. Q–R: Serum levels of ALT (Q) and AST (R) of mice. n = 6. S: Hepatic TG levels in mice. n = 6. For statistical analysis, data are presented as mean ± SEM. Two-way ANOVAs and Unpaired t-tests were performed. ∗*P* < 0.05, ∗∗*P* < 0.01, ∗∗∗*P* < 0.001.

To further explore the effect of SMURF2 in regulating the metabolism of obese mice. AAV-Smurf2 was used to overexpress SMURF2 in adipose of obese mice which has been with HFD feeding for 12 weeks (Fig. 5A). Although body weight showed only a modest reduction in AAV-Smurf2 mice compared to controls (Supplemental Fig. S5A). Fat mass was significantly decreased, including eWAT and sWAT depots (Fig. 5B, C). Remarkably, AAV-Smurf2 mice showed significant improvements in systemic glucose homeostasis by GTT and ITT (Fig. 5D, E). Reduced serum levels of FFA, TG, and T-CHO were also detected in AAV-Smurf2 mice (Fig. 5F–H). Smaller adipocytes, reduced F4/80^+^ macrophage infiltration and αSMA^+^ fibrotic areas were observed in sWAT and eWAT sections of AAV-Smurf2 mice (Fig. 5I, J and Supplemental Fig. S5B). Adipogenic genes were upregulated (Fig. 5K and Supplemental Fig. S5C), while fibrotic and inflammatory genes were suppressed in both sWAT and eWAT of SMURF2 overexpressing mice (Fig. 5L and Supplemental Fig. S5D, E). The expression of thermogenic genes was also not affected in sWAT of SMURF2 overexpressing mice (Supplemental Fig. S5F). SMURF2 overexpression obviously inhibited the p-Smad2 level in eWAT of mice (Fig. 5M), which indicates a robustly weakened TGF-β signaling. Improved hepatic steatosis, decreased F4/80 and Sirius Red content were found in the liver of SMURF2 overexpressing mice (Fig. 5N). Inflammatory and fibrotic gene expression was also inhibited in the liver of SMURF2 overexpressing mice (Fig. 5O, P). Serum ALT and AST levels were markedly reduced in SMURF2 overexpressing mice (Fig. 5Q, R). TG content in the liver was also lower in SMURF2 overexpressing mice (Fig. 5S). Together, these results demonstrate that SMURF2 overexpression significantly alleviates metabolic disorders of obese mice.

### Pharmacological inhibition of SMAD2 phosphorylation by a peptide improves systemic metabolism of obese mice

**Fig. 6:**
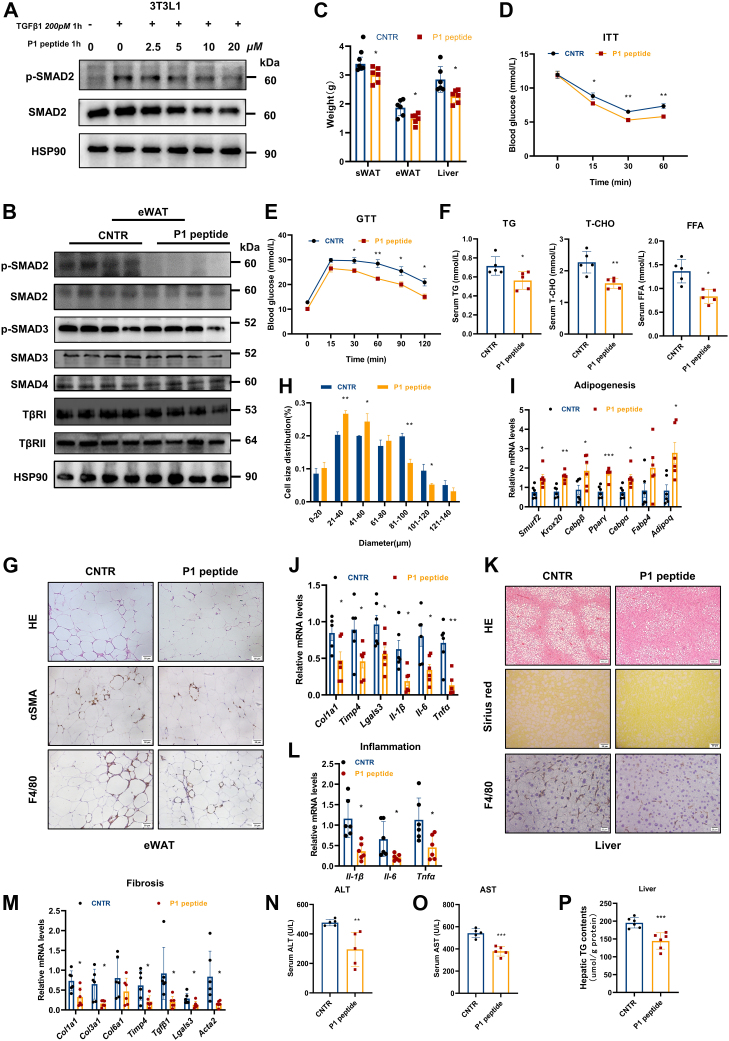
Blocking Phosphorylation and Ubiquitination of SMAD2 by a Peptide Ameliorates Metabolic Dysfunction in Obese Mice. A: Effects of different concentrations of P1 peptide on p-SMAD2 and SMAD2 protein levels in 3T3-L1 cells with 200 *pM* TGFβ1. B: 8-week-old male mice were injected intraperitoneally with P1 peptide or saline for 3 weeks (8 mg/kg body weight, three times per week). Protein levels of p-SMAD2, SMAD2, p-SMAD3, SMAD3, SMAD4, TβRI, and TβRII in eWAT were analyzed by Western blot. C–R: Male C57BL/6J mice were fed an HFD for 12 weeks and then treated with intraperitoneal injections of P1 or saline for 6 weeks. C: Tissue weight of P1 peptide or saline treated mice. n = 6. D–E: ITT (D) and GTT (E) analysis for the mice. F: Serum levels of FFA, TG, and T-CHO of the mice. G: Representative H&E, F4/80, and αSMA staining of eWAT of the mice, Scale bar, 50 μm. H: Statistical analysis of adipocyte size in (G). I-J: qRT-PCR analysis of adipogenic genes (I) and pro-fibrotic/pro-inflammatory genes expression (J) in eWAT of mice. n = 6. K: Representative H&E (scale bar, 100 μm), Sirius red (scale bar, 50 μm), and F4/80 (scale bar, 20 μm) staining of liver sections. L–M, qRT-PCR analysis of hepatic inflammatory (L) and fibrotic (M) genes expression. n = 6. N–O: Serum levels of ALT (N) and AST (O) of the mice. n = 6. P, Hepatic TG levels in mice. n = 6. For statistical analysis, data are presented as mean ± SEM. Two-way ANOVAs and Unpaired t-tests were performed. ∗*P* < 0.05, ∗∗*P* < 0.01, ∗∗∗*P* < 0.001.

To explore a potential pharmacological strategy in treating metabolic disorders, we designed a peptide inhibitor (Peptide 1, P1) that specifically blocks SMAD2 phosphorylation, which is described in detail in Materials and Methods. Western Blot results demonstrated that the P1 peptide can significantly inhibit SMAD2 phosphorylation in 3T3L1 and SVF cells, indicating the effective inhibition of P1, and it can be smoothly taken up by cells (Fig. 6A, Supplemental Fig. S6A). Then we verified that the P1 peptide has a strong inhibitory effect on SMAD2 phosphorylation in eWAT of mice (Fig. 6B). We also found that P1 peptide did not significantly affect the protein levels of SMAD3, SMAD4, TβRII, and TβRI (Fig. 6B). We next administered study with this peptide in obese mice. While body weight was comparable between P1 and saline treatment groups (Supplemental Fig. S6B), both adipose tissue and liver weights were reduced, including eWAT and sWAT (Fig. 6C). Importantly, P1 treatment obviously improved glucose tolerance and insulin sensitivity of obese mice (Fig. 6D, E). Serum levels of FFA, TG, and T-CHO were also lower in mice with P1 treatment than in controls (Fig. 6F). WAT sections from P1-treated mice showed a shift toward a metabolically favorable phenotype, adipocytes were smaller and more numerous, with diminished crown-like structures and reduced αSMA and F4/80 staining (Fig. 6G, H and Supplemental Fig. S6C). This was accompanied by upregulation of adipogenic genes and downregulation of fibrotic and inflammatory genes in eWAT and sWAT (Fig. 6I, J and Supplemental Fig. S6D–F). Also, hepatic lipid accumulation, inflammation, and fibrosis were significantly attenuated in P1-treated mice (Fig. 6K–M). Serum ALT and AST levels were markedly reduced in the P1 treatment group (Fig. 6N, O). TG content in the liver was lower in SMURF2 overexpressing mice than that of controls (Fig. 6P). Together, these findings provide a potential method that the P1 peptide is beneficial for treating obesity-associated metabolic disorders.

## Discussion

TGF-β acts as a "fuel additive" in the development of obesity and its complications. Our study reveals a pathway to mitigate the detrimental effects of TGF-β in adipose during obesity. E3 ligase SMURF2 plays a role in promoting adipogenesis and ameliorating obesity-related metabolic diseases by inhibiting TGF-β/SMAD signaling. This work provides evidence that SMURF2 modulates adipose expansion preferentially via the formation of new adipocytes while suppressing pathological adipocyte hypertrophy, thereby significantly improving adipose dysfunction caused by energy surplus. Intervention targeting SMAD ubiquitination to counteract TGF-β-induced physiological dysfunction in adipose markedly improves systemic insulin resistance, fatty liver disease, and other obesity-related complications, offering a potential therapeutic target for translational research.

Ubiquitination of SMAD2/3 is crucial for regulating TGF-β signaling (14, 26). This study innovatively propose that SMAD ubiquitination is a key mechanism driving early-stage adipogenic differentiation. We discovered a moderate decrease in p-SMAD2/3 during adipogenic differentiation, while total SMAD2/3 levels remained unchanged at both protein and RNA levels. Importantly, the proteasome inhibitor MG132 preserved the p-SMAD protein levels, indicating that SMAD protein abundance is closely related to the ubiquitin-proteasome system. It was reported that ubiquitination of p-SMAD2/3 is primarily regulated by the ubiquitin ligase NEDD4L. Our results did not support this mechanism in adipocytes, as NEDD4L did not regulate either SMAD protein levels or the adipogenic process. Another E3 ligase WWP1 was proved to regulate adipogenesis by mediating both poly-ubiquitination and proteasomal degradation of KLF2 (27). Finally, we identified SMURF2 as the crucial ligase regulating the ubiquitination and degradation of p-SMAD2. Notably, SMURF2 mainly mediates the ubiquitination and degradation of p-SMAD2 while mono-ubiquitination for SMAD3 without directly affecting SMAD3 stability. The altered level of p-SMAD3 seems likely because p-SMAD2/3 functions as a protein complex, and degradation of p-SMAD2 affects the stability of the complex (28, 29).

SMURF2 has also been shown to play multiple roles and mechanisms in diseases such as tumors and fibrosis. The role of SMURF2 in cancer is dural which is highly dependent on the substrate specificity. On the one hand, SMURF2 promotes the degradation of oncogenic proteins in TGF-β pathways such as TβRI and SMAD2, inhibiting cancer cell proliferation and metastasis. On the other hand, SMURF2 stabilizes proteins such as KRAS through K63 ubiquitination or indirectly activates KRAS by degrading GAP17-1 (30). SMURF2 typically plays a "brake" role in inhibiting fibrosis diseases. In liver fibrosis tissue, the expression level of SMURF2 is significantly reduced, and the degradation of TβRI is weakened, leading to excessive activity of TGF-β signaling and worsening fibrosis (31). In endometrial fibrosis (intrauterine adhesions), SMURF2 stabilizes the inhibitory SMAD protein SMAD6 by catalyzing K63 linked polyubiquitination. Stable SMAD6 can more effectively inhibit the TGF - β signaling pathway, reduce collagen deposition, and thus alleviate fibrosis (32). The role of SMURF2 in pulmonary fibrosis is slightly different. Research has found that the expression of SMURF2 is upregulated in silicosis fibrosis. It activates the TGF - β signaling pathway by degrading the inhibitory protein SMAD7, thereby promoting epithelial–mesenchymal transition (EMT) and fibrosis progression (33). Our research complements the novel role of SMURF2 as a ubiquitin ligase in regulating adipogenesis and lipid metabolism.

During adipogenic differentiation of preadipocytes, the time course of SMURF2 expression coincides with SMAD protein levels. This is characterized by increased SMURF2 and decreased SMAD, both occurs almost at the same period. This suggests that when adipocyte precursors initiate differentiation, upregulation of SMURF2 inhibits the TGF-β/SMAD braking signal, thereby sustaining the physiological process of adipogenesis. At the cellular level, overexpression or knockdown of SMURF2 significantly promoted or inhibited adipocyte precursor differentiation, respectively. In animal models, overexpression of SMURF2 in mice markedly increased the population of newly formed adipocytes in adipose tissue. Collectively, our findings demonstrate that SMURF2 promotes adipogenesis by inhibiting the TGF-β/SMAD braking signal, which plays a crucial role in driving adipogenic differentiation.

The role of SMURF2 in ubiquitinating SMAD and inhibiting TGF-β signaling in adipose suggests its importance in ameliorating obesity and systemic metabolism. We detected significantly lower expression of SMURF2 in the adipose tissue of obese mice compared to normal controls. This was also observed in human samples and databases, further supporting a close association between SMURF2 and obesity. In SMURF2-overexpressing mice subjected to a high-fat diet, adipose tissue exhibited a pronounced increase in newly formed adipocytes and a significant reduction in pathologically hypertrophic mature adipocytes. The healthy adipose lead to substantial whole-body metabolic benefits (34, 35). In a therapeutic model, increasing SMURF2 levels in adipose tissue of obese mice significantly alleviated adipose inflammation and fibrosis, with marked improvements in systemic insulin resistance and fatty liver disease. These beneficial effects of SMURF2 on obesity progression and treatment are closely related to diminished TGF-β pathway activity.

In recent years, significant progress has been made in the research and development of drugs targeting ubiquitin-related targets (36, 37, 38). Based on our findings, we reasoned that targeting SMAD ubiquitination could ameliorate obesity and metabolic disorders. We designed a small peptide targeting the SMAD2 phosphorylation site for inhibitory intervention. Administration of this peptide to high-fat diet-induced obese mice resulted in satisfactory metabolic improvements. Although no significant reduction in body weight was observed in a short period, glucose tolerance and fatty liver disease were markedly improved. Due to the wide-ranging effects of the TGF-β pathway (39), the intraperitoneal systemic administration method we used in our study is not the most appropriate. Fortunately, the enhancement of the TGF - β/SMAD signaling pathway mainly produces toxic side effects on the body's metabolism, including the liver and skeletal muscle (40, 41). Therefore, the regulation of overall metabolism in mice by P1 peptide is mainly beneficial. Although we did not observe any significant side effects in our study, it would be safer if the inhibitor could directly target the adipose tissue. This research aims to provide important theoretical and experimental foundations to open up new ideas and methods for obesity-related metabolic diseases.

## Data Availability

All data needed to evaluate the conclusions in the paper are present in the paper and/or the Supplementary Materials.

## Supplemental data

This article contains supplemental data.

## Conflict of interest

The authors declare that they have no conflicts of interest with the contents of this article.
