# Supplementary material for: E3 ligase SMURF2 promotes adipogenesis and improves obesity complications by suppressing TGF-β signaling
Source: J Lipid Res. 2026 May 18;67(7):101061. doi: 10.1016/j.jlr.2026.101061 (PMC13320998; doi:10.1016/j.jlr.2026.101061)
Supplement: Supplementary Figures [file mmc1.pdf]

SUPPLEMENTAL INFORMATION

Fig. S1

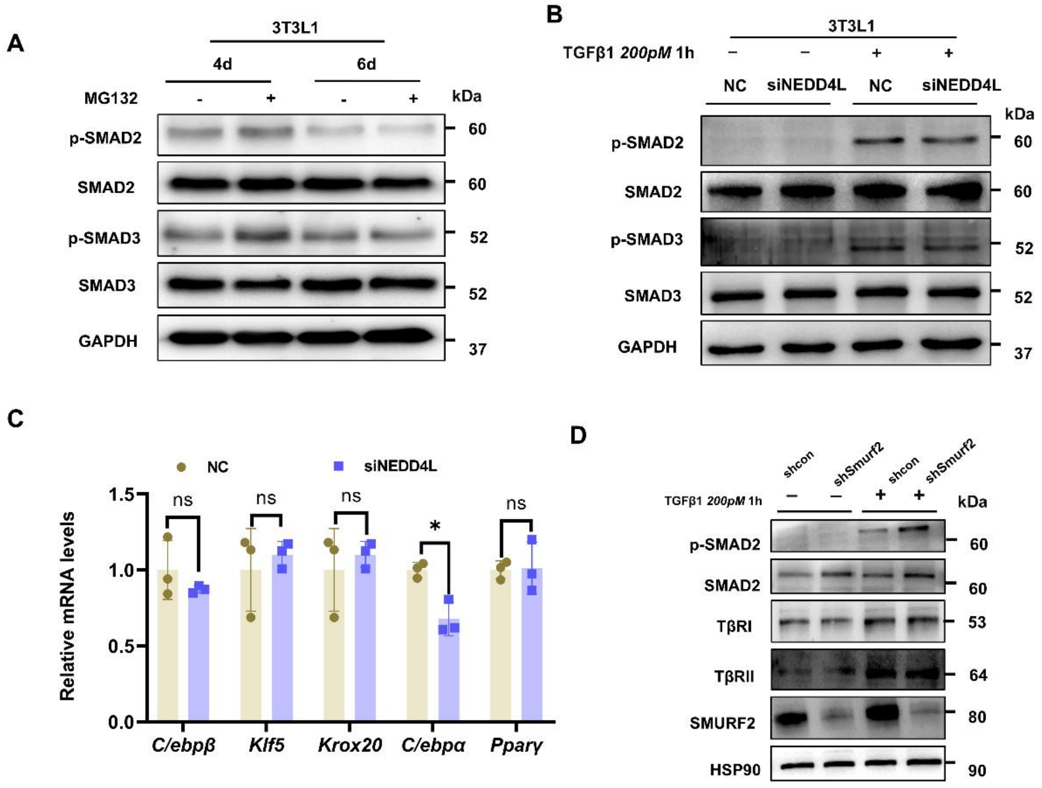

Fig. S1 NEDD4L is Dispensable for SMAD2/3 Ubiquitination in Adipocytes.

A, Western blot analysis of SMAD2/3protein levels during adipogenic differentiation of 3T3-L1 cells at day 4 and 6, cells were treated with 20 μM MG132 for 4h before harvest. **B,** Protein levels of SMAD2/SMAD3 in 3T3-L1 cells with NEDD4L knockdown, cells were treated with 200 pM TGFβ1 for 1 hour before harvest. **C,** mRNA levels of adipogenic genes during adipocyte differentiation of 3T3-L1 cells with NEDD4L knockdown. **D,** Protein levels of p-SMAD2/SMAD2, TβRI, and TβRII in 3T3-L1 cells with SMURF2 knockdown, cells were treated with vehicle or 200 pM TGFβ1 for 1 hour before harvest. Statistical data are presented as mean ± SEM, and statistical analysis was performed using an unpaired t-test. *P<0.05. n=3.

Fig. S2

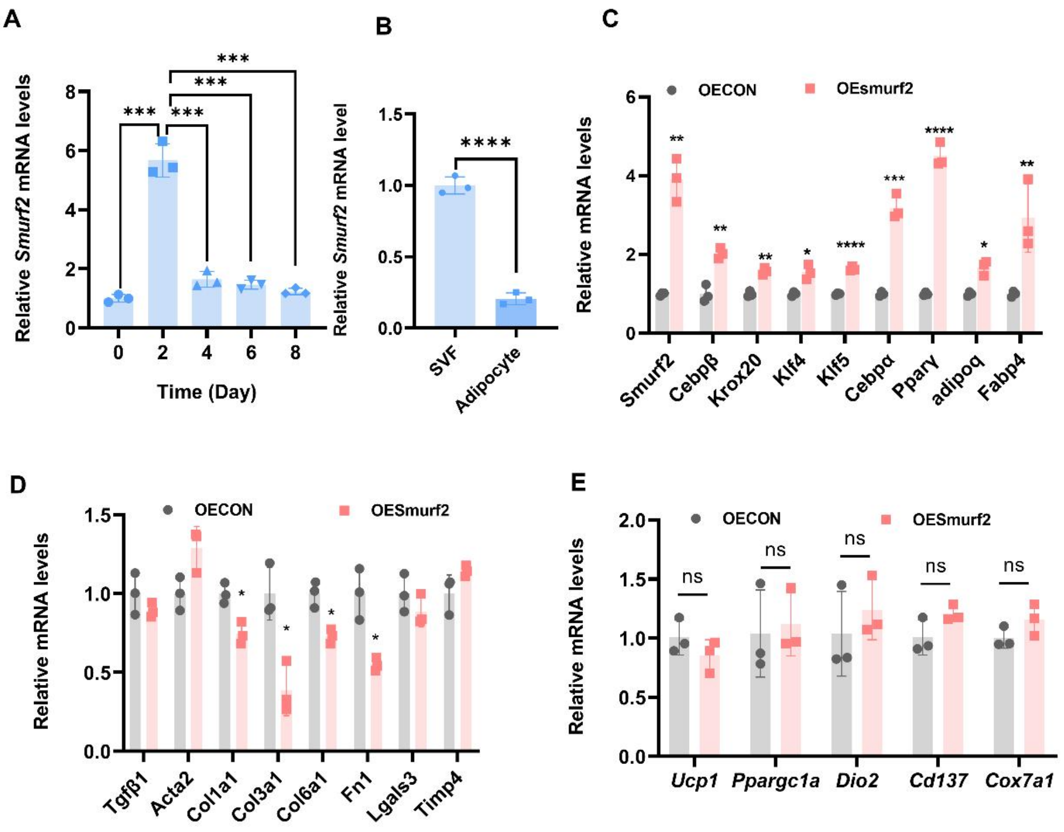

Fig. S2 SMURF2 Promotes Adipogenic Genes Expression *in vitro*.

A, Smurf2 mRNA levels during adipogenic differentiation of SVF cells at days 0, 2, 4, 6 and 8. **B,** Relative Smurf2 mRNA levels in SVF and mature adipocyte. **C,** mRNA levels of adipogenic genes in 3T3-L1 cells with Smurf2 overexpression on day 6 of adipocyte differentiation. **D,** mRNA levels of fibrosis genes of 3T3-L1 cells after Smurf2 overexpression with TGFβ1 treatment (200 pM, 1h), analyzed by qRT-PCR. **E,** mRNA levels of thermogenic genes of 3T3-L1 cells after Smurf2 overexpression. For statistical analysis, data are presented as mean ± SEM. One-way ANOVA test was performed in A. Unpaired t-tests were performed in B-E. *P<0.05, **P<0.01, ***P<0.001, ****P<0.0001. n=3.

Fig. S3

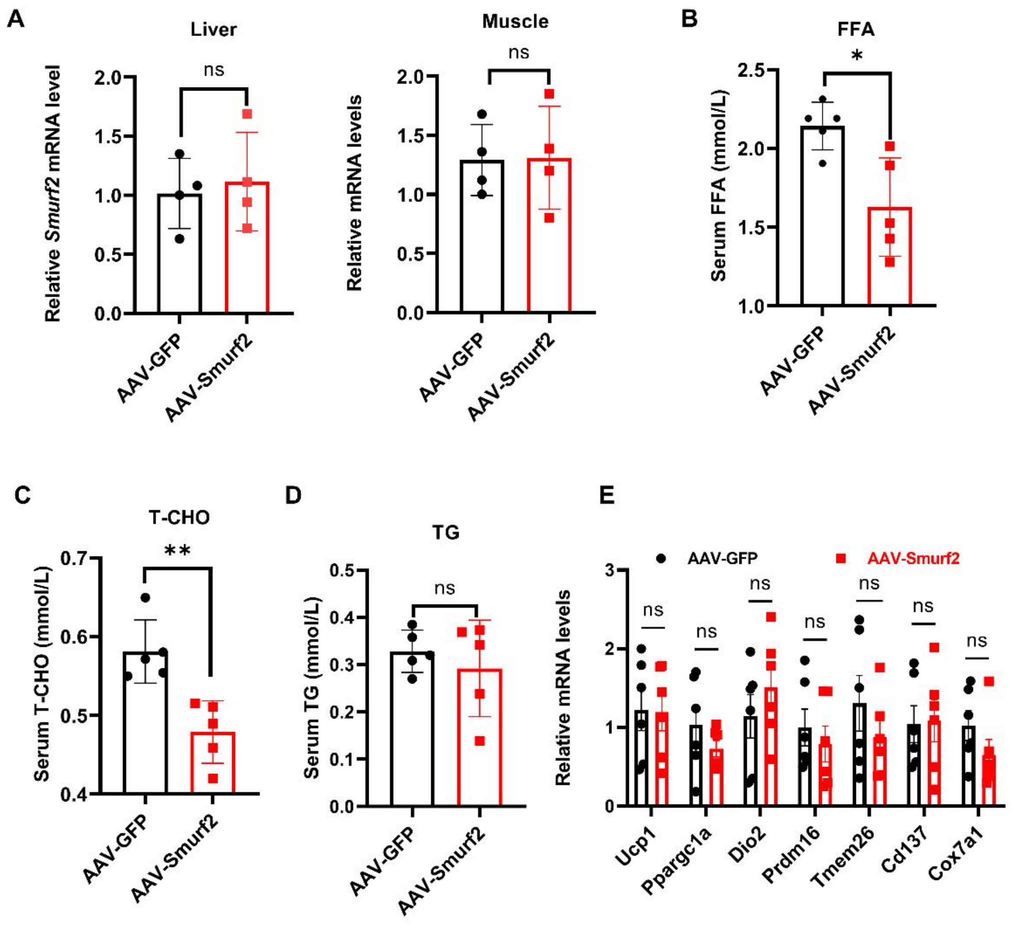

Fig. S3 SMURF2 Overexpression Reduces FFA and T-CHO in Mice.

A, Smurf2 mRNA levels of liver and muscle in AAV-GFP and AAV-Smurf2 mice, n=4. **B-D,** Serum free fatty acids (FFA) (B), total cholesterol (T-CHO) (C) and triglycerides (TG) (C) levels of AAV-GFP and AAV-Smurf2 mice fed with NCD for 12 weeks, n=5. **E,** mRNA levels of thermogenic genes of sWAT in AAV-GFP and AAV-Smurf2 mice, n=5. For statistical analysis, data are presented as mean ± SEM. Unpaired t-tests were performed. *P<0.05, **P<0.01.

Fig.S4

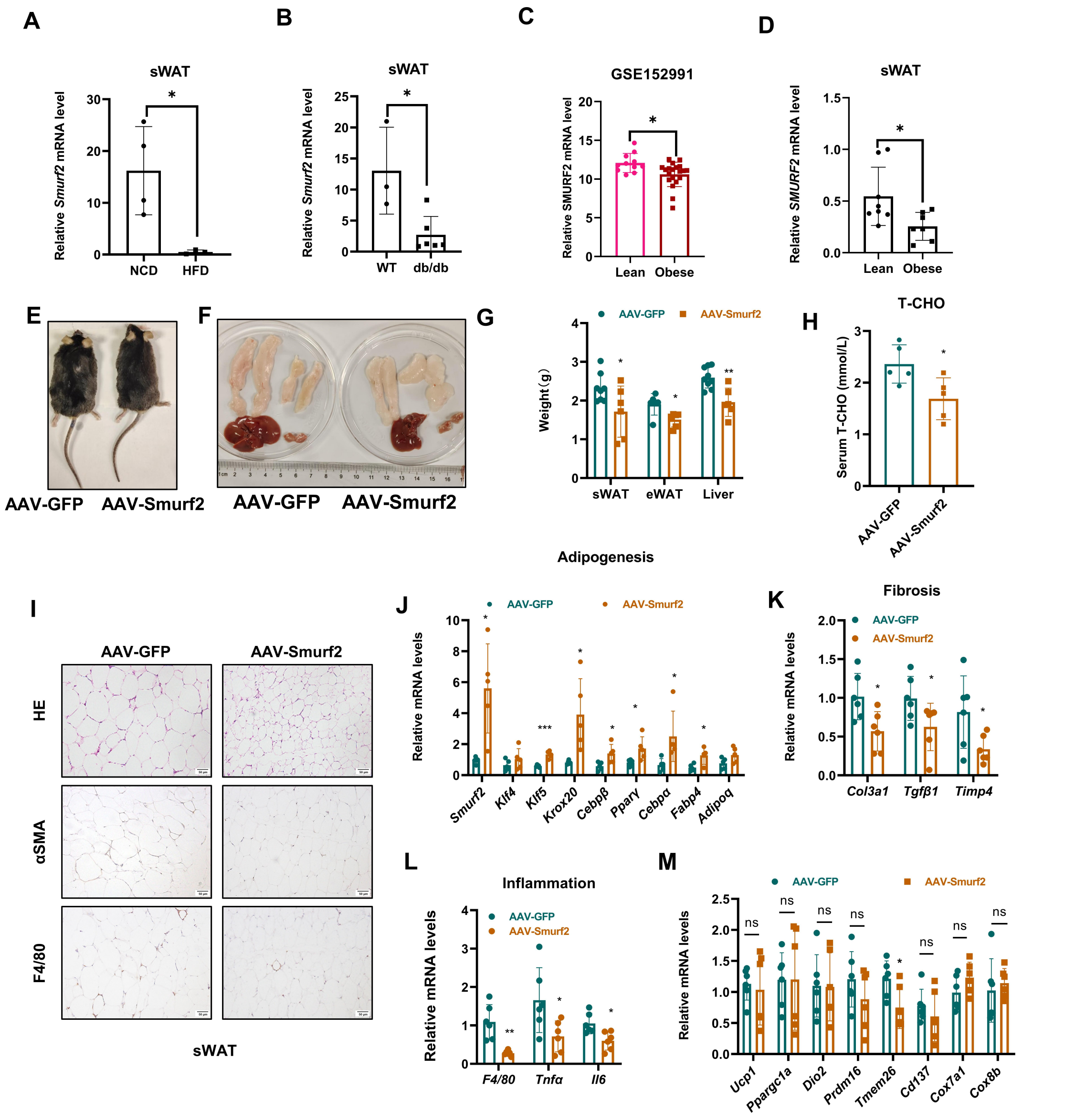

Fig.S4 SMURF2 Overexpression Attenuates Adipose Fibrosis and Inflammation in Obesity.

A-B, qRT-PCR analysis of SMURF2 mRNA levels in sWAT of mice fed a high-fat diet (HFD) vs. chow diet (A), and in db/db vs. wild-type (WT) mice (B). **C-D,**Analysis of SMURF2 mRNA expression in human sWAT from public datasets (GSE152991) (C) and in an independent cohort of sWAT samples from lean and obese individuals (D). **E,** Representative photographs of AAV-GFP and AAV-Smurf2 mice fed with HFD for 12 weeks,

n=8. **F,** Representative photographs of subcutaneous adipose tissue (upper left), liver (lower left), epididymal adipose tissue (upper right) and and brown adipose tissue (lower right) from AAV-GFP and AAV-Smurf2 mice fed with HFD for 12 weeks, n=8. **G,** Tissue weight of AAV-GFP and AAV-Smurf2 mice fed with HFD for 12 weeks, n=6. **H,** Serum levels of T-CHO of the mice, n=6. **I,** Representative HE, F4/80 and αSMA immunohistochemical staining of sWAT from the mice, scale bar, 50 μm.**J-L,** qRT-PCR analysis of adipogenic genes (J) pro-fibrotic(K) and pro-inflammatory genes (L) expression in sWAT, n=6. **M,** mRNA levels of thermogenic genes in sWAT. Statistical data are presented as mean ± SEM, and statistical analysis was performed using an unpaired t-test. *P<0.05, **P<0.01, ***P<0.001.

Fig.S5

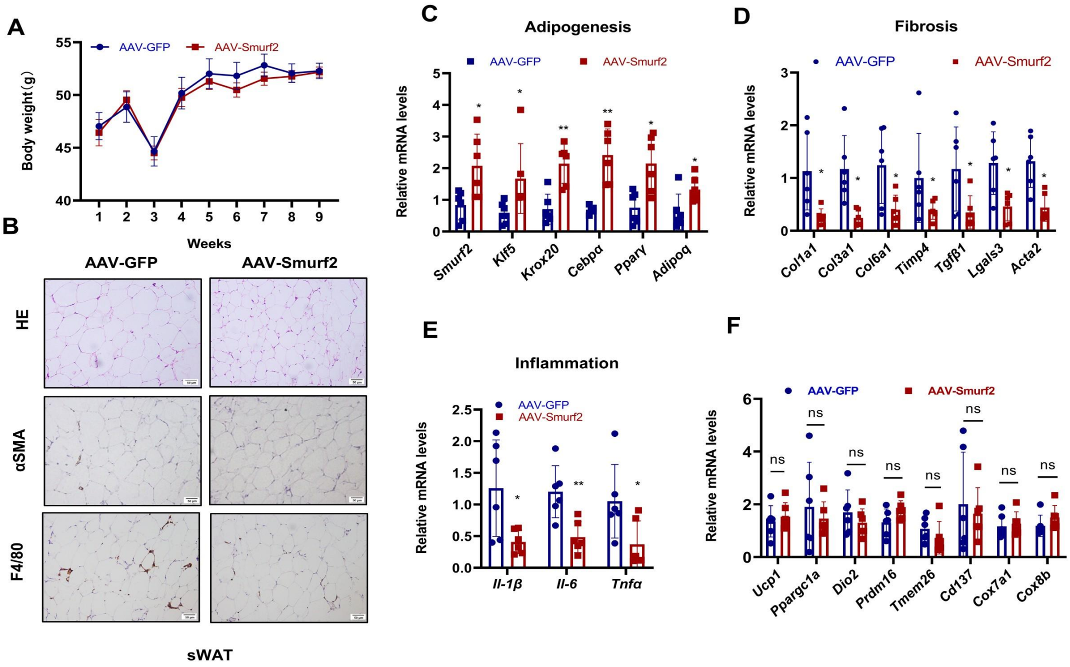

Fig.S5 SMURF2 Overexpression in Obese Mice Promotes Adipogenesis and Suppresses Inflammation.

A, Body weight curve of the mice. n=8.**B,** Representative H&E, F4/80, and αSMA staining of sWAT sections of the mice, Scale bar, 50μm.**C-F,** qRT-PCR analysis of adipogenic genes (C), pro-fibrotic genes (D), pro-inflammatory genes (E) and thermogenic genes expression (F) in sWAT, n=6. Statistical data are presented as mean ± SEM, and statistical analysis was performed using an unpaired t-test. *P<0.05, **P<0.01, ***P<0.001

Fig. S6

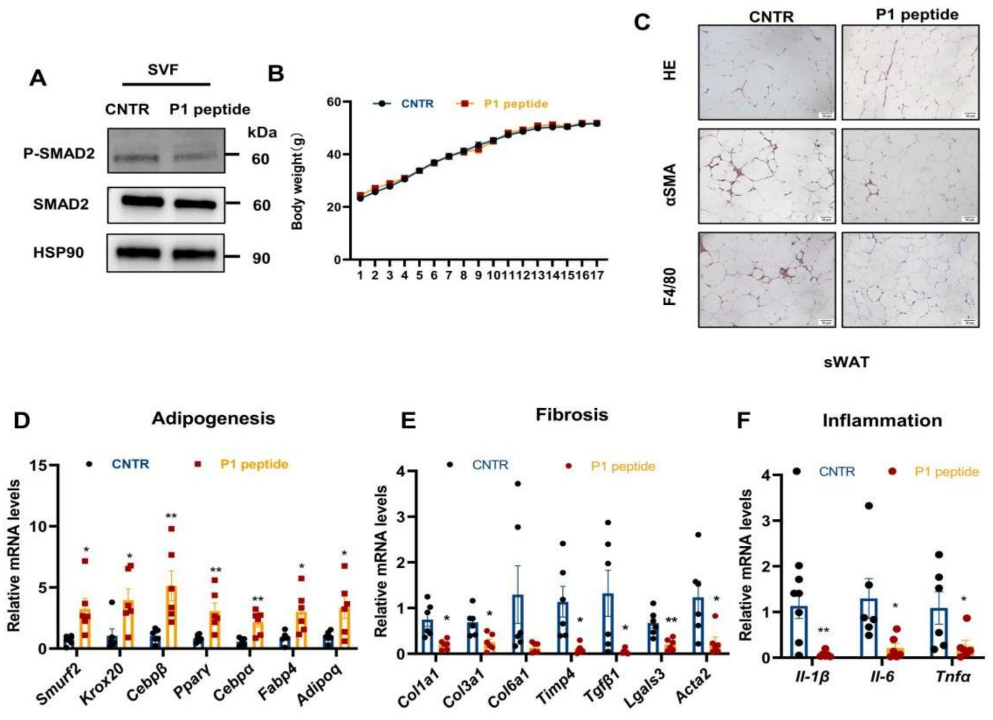

Fig. S6 P1 Peptide Administration Improves Metabolic Health of Obese Mice.

A, Protein levels of p-SMAD2 and SMAD2 in SVF from mice treated with P1 peptide or saline for 3 weeks. **B,** Body weight curve of the mice, n=8. **C,** Representative H&E, F4/80, and αSMA staining of sWAT of the mice, Scale bar, 50μm. **D-F,** qRT-PCR analysis of adipogenic genes (D) and pro-fibrotic (E) and pro-inflammatory genes expression (F) in sWAT, n=6. Statistical data are presented as mean ± SEM, and statistical analysis was performed using Two-way ANOVAs and unpaired t-test. **P*<0.05, ***P*<0.01, ****P*<0.001.
