# Supplementary material for: E3 ligase SMURF2 promotes adipogenesis and improves obesity complications by suppressing TGF-β signaling
Source: J Lipid Res. 2026 May 18;67(7):101061. doi: 10.1016/j.jlr.2026.101061 (PMC13320998; doi:10.1016/j.jlr.2026.101061)
Supplement: Supplementary Table [file mmc2.pdf]

Supplementary Table S1 Primer sequence table

Genes	Forward	Reverse
	**Mus musculus**	
β-actin	ACCTTCTACAATGAGCTGCG	CCTGGATAGCAACGTACATGG
SMURF2	CATCAACCGCCTCAAGGACA	CCTATTCGGTCTCTGGACTGAAG
C/EBPβ	TGGACAAGCTGAGCGACGAG	GCTGCTCCACCTTCTTCTGC
Klf5	TTCGCCAACTCTCCCACCTG	TGCACTTGTAGGGCTTCTCG
Klf4	GCACACCTGCGAACTCACAC	AGGCCCTGTCACACTTCTGG
KROX20	CTCGTCGGTGACCATCTTCC	TCGGATACGGGAGATCCAGG
C/EBPα	GACATCAGCGCCTACATCGA	TCGGCTGTGCTGGAAGAG
PPARγ	GAAAGACAACGGACAAATCACC	GGGGGTGATATGTTTGAACTTG
Adiponectin	GGAGAGAAAGGAGATGCAGGT	CTTTCCTGCCAGGGGTTC
FABP4	TTTACAAAATGTGTGATGCCTT	TAAAAGTACTCTCTGACCGGAT
Timp4	CTTGGTTCCCTGGCGTACTC	ACCTGATCCGTCCACAAACAG
Col1a1	TGCTAACGTGGTTCGTGACCGT	ACATCTTGAGGTCGCGGCATGT
Col3a1	ACGTAAGCACTGGTGGACAG	CCGGCTGGAAAGAAGTCTGA
Col6a1	AACAGGAATAGGAAATGTGACCC	ACACCACGGATAGGTTAGGGG
Tgf1β	ATTTGGAGCCTGGACACACA	GAGCGCACAATCATGTTGGA
Acta2	CCCAGACATCAGGGAGTAATGG	TCTATCGGATACTTCAGCGTCA
Lgals3	CCCTTTGAGAGTGGCAAACCA	CATCGTTGACCGCAACCTT
Tnfa	AGGGTCTGGGCCATAGAACT	CCACCACGCTCTTCTGTCTAC
F4/80	CTTTGGCTATGGGCTTCCAGTC	GCAAGGAGGACAGAGTTTATCGTG
Il1b	AATCTCGCAGCAGCACATCAAC	TGTCCTCATCCTGGAAGGTC
Il6	GGATACCACTCCCAACAGACC	GCAAGTGCATCATCGTTGTTC
	**Homo sapiens**	
HSP90	GTCTGTGAAGGATCTGGTCATC	CAGCAGTAGGGTCATCTTCATC
SMURF2	ACACTGGCTACCAGCGTTTG	TCTGTCTCGGGTCTGTAAACTG

Supplementary Table S2. Primary Antibodies List

Smurf2(D8B8)	12024	CST
Smad2 Rabbit mAb	19114	ABclonal
Phospho-Smad2-T220 Rabbit pAb	AP0909	ABclonal
Phospho-Smad3-T179 Rabbit pAb	AP0554	ABclonal
Smad3 Rabbit pAb	(A16913)	ABclonal
SMAD4 (D3R4N)	46535	CST
PPARγ(81B8)	71433	CST
C/EBPα(PT0665R)	YM8474	Immunoway
C/EBPβ	YT0553	Immunoway
TGFβ R1	sc-101574	SANTA
TGFβ R11	sc-17792	SANTA
HSP90	13171-1-AP	Proteintech
GAPDH	5174	CST
Rabbit Anti-Mouse IgG	ab6728	abcam
Goat Anti-Rabbit IgG	ab6721	abcam
